# Analysis of the clinical and epidemiological profile of leprosy in Brazil and Major Regions^⋆^

**DOI:** 10.1016/j.abd.2025.501127

**Published:** 2025-06-18

**Authors:** Alícia de Siqueira Sczmanski, Lucas Pazin, Thiago Mamoru Sakae, Josete Mazon

**Affiliations:** Centro de Ciências, Tecnologias e Saúde, Universidade Federal de Santa Catarina, Araranguá, SC, Brazil

**Keywords:** Leprosy, Leprosy/epidemiology, Neglected diseases, Neglected diseases/epidemiology

## Abstract

**Background:**

Leprosy is an infectious disease, endemic to Brazil, associated with poor living conditions. Although curable, it is a neglected disease, posing a serious public health problem. The literature lacks comprehensive and recent analyses of the pathology.

**Objectives:**

The study aims to describe and analyze the clinical-epidemiological profile of leprosy patients in large Brazilian regions and compare it with regional sociodemographic indicators.

**Methods:**

This is an ecological, descriptive, and analytical observational study, carried out through the collection of secondary data from the Disease Notification Information System from 2014 to 2019, the 2010 census, and population estimates from the Brazilian Institute of Geography and Statistics.

**Results:**

A total of 215,155 new cases were reported and the annual detection rate was maintained in four Brazilian regions, with only the South showing a significant decrease. The highest rate was observed in the Midwest and the lowest in the South. The predominant profile was male, age range between 60 and 79 years old, literate, and black and brown ethnicity. There was a predominance of the borderline cases, multibacillary form, more than five skin lesions, and zero grade of disability at diagnosis. Bacilloscopy was not performed in 42.8% of the cases and cure was the outcome in 77%.

**Study limitations:**

The use of secondary data and the time interval analyzed are restricted by the data source platform and the COVID-19 pandemic.

**Conclusions:**

This shows a perpetuated public health problem, mainly in the North, Northeast, and Midwest regions, associated with socioeconomic indicators and has a clinical-epidemiological profile that favors the bacillus transmission.

## Introduction

Leprosy is characterized as an infectious-contagious disease caused by *Mycobacterium leprae* (*M. leprae*), known as Hansen's bacillus, and *Mycobacterium lepromatosis* (*M. lepromatosis*). It is one of the oldest diseases known to humanity, with reports dating back over 3,000 years. The etiological agent was first described in 1873 by Norwegian physician Gerhard Armauer Hansen.^1^

The parasite is an acid-fast, obligate intracellular bacillus, which multiplies slowly and does not proliferate *in vitro*, making it difficult to obtain scientific knowledge about it.^2^ Despite its low pathogenicity, the bacillus is highly infective,^3^ has an affinity for skin cells and peripheral nerves, and its multiplication time is slow, lasting 11 to 16 days.^3^ It is estimated that the disease incubation period lasts an average of five years.^2^ The infection is mainly transmitted by untreated sick individuals with a high bacillary load, who excrete the bacillus through the upper airways, which also act as a gateway for susceptible individuals. Transmission occurs through direct contact between individuals and is facilitated by the coexistence of susceptible individuals with untreated patients. The hematogenous route is the mechanism of dissemination to the skin, nerves, mucous membranes and other tissues.^2^ Susceptibility to the bacteria has a strong genetic influence so family members of individuals with leprosy are more likely to become ill.

It manifests itself through marked dermatoneurological signs and symptoms such as skin and peripheral nerve lesions. There are four clinical forms described: indeterminate, tuberculoid, lepromatous, and borderline, with the latter being subdivided into borderline-tuberculoid, borderline-borderline, and borderline-lepromatous. There is also the primary or pure form of neural leprosy, which does not show skin lesions and, although it is not listed as a specific clinical form in the pathology notification form, represents up to 10% of all cases.^3^ The disease is severe, and the infected individual may develop various degrees of physical disabilities and deformities, often irreversible ones, which lead to several problems in the social, professional, and psychological spheres.^3^ However, since the disease has well-defined peculiarities, diagnosis is simple in most cases: it is made through anamnesis and physical examination with or without the aid of a bacilloscopy test^2^ and a correct and early diagnosis allows the cure and interruption of the disease transmission chain. Treatment is effective and available through the Brazilian Unified Health System.^2^

It is a disease almost exclusive to the developing world, associated with poor socioeconomic conditions, poverty, and lack of access to adequate housing, food, health and education.^2^ It is classified as a neglected disease that affects areas of Asia, Africa and Latin America,^4^ and three countries account for approximately 80% of cases: India (56.6%), Brazil (13.8%) and Indonesia (8.6%). Leprosy is an endemic disease in Brazil, a public health problem that needs to be overcome. The country is responsible for more than 90% of new cases of infection in the entire region of the Americas. As part of the National List of Compulsory Notification of Diseases, Injuries and Public Health Events (MS/GM Consolidation Ordinance Number 4, of September 28, 2017), 215,155 new cases were diagnosed between 2014 and 2019.^5^, ^6^

Given Brazil's position in terms of prevalence in the global panorama, it is clear that studies with current data need to be carried out, seeking to outline the current clinical and epidemiological profile of leprosy. As the disease incidence has a positive association with socioeconomic factors,^7^ it is essential to correlate epidemiology to the social indicators of each region, since the country has continental dimensions, divided into areas with different climatic and social conditions.

Thus, this study sought to compare the clinical-epidemiological profile of leprosy in different regions of Brazil, according to sex, age group, skin color or race and illiteracy; to analyze the distribution of leprosy in the five Brazilian regions and to correlate the annual detection rate of leprosy with socioeconomic indicators of the regions.

## Methods

### Study design and area

This is an observational, ecological, descriptive and analytical study, using secondary data collection. The research population consisted of new cases of leprosy diagnosed and registered on the SINAN platform using the TABNET tool from 2014 to 2019. This interval was delimited in view of the update of SINAN and TABNET in 2014, which aimed to improve data quality, so that previous data are subject to inaccuracy, and due to the distortions caused by the COVID-19 Pandemic regarding the number of new cases in 2020 and 2021.

### Data source and variables

In this study, data from patients residing in Brazil whose new diagnosis was registered on SINAN and TABNET from 2014 to 2019 were used for the temporal analysis. All cases that did not contain data of interest for the research were excluded.

Data collection was carried out virtually, on the SINAN website on the page “Indicators and Basic Data on Leprosy in Brazilian Municipalities” ‒ http://indicadoreshanseniase.aids.gov.br, where data from SINAN, the Ministry of Health (MoH) and the Health Surveillance Secretariat (SVS, *Secretaria de Vigilância em Saúde*) can be found, and on the DATASUS website, through the TABNET page “Leprosy Cases – Since 2001 (SINAN)” ‒ http://tabnet.datasus.gov.br/cgi/tabcgi.exe?sinannet/cnv/hanswbr.def. Then, the five Brazilian regions and the variables of interest were selected: sex of the individual, age group, skin color or race, illiteracy, clinical form, operational classification, bacilloscopy, skin lesions, type of outcome, grade of disability and year of diagnosis.

The population and socioeconomic data for each region were obtained from the most recent complete national census available, currently from 2010, found on the Brazilian Institute of Geography and Statistics (IBGE, *Instituto Brasileiro de Geografia e Estatística*) 2010 Census website ‒ https://censo2010.ibge.gov.br, and also from the most recent National Household Sample Survey (PNAD, *Pesquisa Nacional por Amostra de Domicílio*), also found on the IBGE website ‒ https://www.ibge.gov.br/estatisticas/sociais/populacao/9127-pesquisa-nacional-por-amostra-de-domicilios.html?=&t=resultados.

The data were transformed into an annual detection rate of new cases for the total cases and an average detection rate between 2014 and 2019 for the analyzed variables, all calculated per 100,000 inhabitants, for Brazil and its Major Regions.

(1) Anual detection rate = (Number of New Cases/Total Resident Population) × 100,000

It is noteworthy the impossibility of calculating the average detection rate of new cases according to skin color or race in the “unknown/white” classification due to the lack of data for this population in the Census.

### Statistical analysis

The data were organized using Google Sheets, and the distribution of variables was obtained in absolute values ​​and proportions (%). The case frequency was transformed into an annual and average detection rate, according to the variables of interest in the study, to compare the incidence between each macro-region and Brazil. The Relative Risk (RR) was calculated for the variables sex, race and illiteracy according to the detection rates.

The statistical analysis was performed using the IBM SPSS software, version 23.0 (SPSS 23.0®). Temporal trends were analyzed using the average annual variation of each category, annual increase rate, linear regression (beta coefficient) and Spearman's correlation coefficient. The investigation of an association between the variables was performed using linear regression and Spearman's correlation. Bivariate analysis with calculation of R² (correlation coefficient) was used for the results of the temporal series. Values ​​of p < 0.05 were considered statistically significant.

(2) Anual increase rate = (Current Year Value - Previous Year Value ) / (Previous Year Value) × 100

## Results

Brazil is the fifth most populous country in the world, with an estimated population of 204,860,101 inhabitants (inhab) according to a survey carried out in 2015 by the Brazilian Institute of Geography and Statistics (IBGE) and has an average Human Development Index (HDI) of 0.724, according to the latest data from the Institute of Applied Economic Research, calculated in 2010. The country is divided into five major regions. The North Region has an estimated population of 17,523,777, and an average HDI of 0.667. The Northeast Region has an estimated population of 56,640,710 and the lowest average HDI in the country - 0.663. The South Region has an estimated population of 29,290,154 and an average HDI of 0.754. The Southeast Region, the most populous and most developed one, has an estimated population of 85,916,158 and an average HDI of 0.766. The Midwest Region has the smallest population among the regions, estimated at 15,489,302, and an average HDI of 0.757.

In the period from 2014 to 2019, a total of 215,155 new cases of leprosy were reported in Brazil (Table 1), with 41,666 cases in the North, 91,623 in the Northeast, 44,988 in the Midwest, 29,855 in the Southeast and 6,976 in the South. The highest annual detection rate of new cases nationwide occurred in 2018 with 17.94/100,000 inhabitants, and the lowest in 2016, at 15.78/100,000 inhabitants.

**Table 1:** Annual detection rate of new cases of leprosy per 100,000 inhabitants. Brazil and Regions, 2014‒2019.

Year/Region	Brazil	North	Northeast	Southeast	South	Midwest
2014	18.76	41.76	29.07	6.39	4.48	50.73
2015	17.59	37.98	27.66	5.99	4.45	46.99
2016	15.78	36.72	24.32	5.59	3.8	40.03
2017	17.04	38.57	27.15	5.56	3.7	44.69
2018	17.94	42.2	26.83	5.54	3.63	53.95
2019	17.85	40.51	26.7	5.68	3.76	54.05
Mean	17.49	39.62	26.95	5.79	3.97	48.41
AI	-0.007	-0.004	-0.014	-0.023	-0.032	0.021
β	-0.119	0.201	-0.397	-0.782	-0.849	0.406
p-value	0.823	0.703	0.436	0.066	0.032	0.425

Fonte: IBGE ‒ DATASUS, adapted by the authors, 2023.

AI, Annual Increase; β, Beta Index.

Table 1 shows the annual detection rate of newly reported cases of leprosy per year during the studied period. There was a slight decline in the detection rate in the Brazilian population from 18.76/100,000 inhab. in 2014 to 17.85/100,000 inhab. In 2019, with a negative Annual Increase (AI) of -0.7%, without statistical significance. Clear differences were observed in the distribution of annual detection rates between the regions of the country, with hyperendemic rates in the Midwest, very high rates in the North and Northeast, and average rates in the Southeast and South. The highest rate observed in the period was 54.05 in 2019 in the Midwest; in contrast, the lowest rate was 3.63 in 2018 in the South. The Midwest was the only region to show a positive AI (2.1%), which shows an average increase. Next, the North (AI -0.4%), Northeast (AI -1.4%), Southeast (AI -2.3%) and, finally, the South (AI -3.2%) with the lowest rate found and a significant decrease in the detection rate (p = 0.032). The other regions showed a trend towards indicator stability (p > 0.05).

In Spearman’s correlation analysis between the variables “year” and “new cases in Brazil”, which was used to calculate the annual detection rate, a negative correlation (-0.086) was observed without statistical significance (p > 0.05). The bivariate analysis with the calculation of *R²* (correlation coefficient = 0.014) for the same two variables also showed no statistical significance. Thus, the correlations demonstrate that the rates remained stable throughout the period.

Regarding the epidemiological characteristics (Table 2), it was observed that the majority of reported cases were male (56.59%), with a relative risk almost 40% higher than females (RR = 1.39; Confidence Interval [95% CI: 1.29‒1.48]; p < 0.00001). The most affected age group was 40‒59 years old, with 62,876 total cases, while the highest detection rate was observed between 60 and 79 years old, with a rate of 28.14/100,000 inhabitants. The main ethnicity is black and brown (71.98%), followed by white (23.24%) and a minority of Asians (1.16%). Regarding the level of schooling, the detection rate in Brazil was higher among illiterate individuals, comprehending 8.99% of the infected population, with a rate of 28.62/100,000 inhabitants, comprising a risk almost 40% higher than that of literate individuals (RR = 1.38; 95% CI: 1.29‒1.45; p < 0.0001).

**Table 2:** Average detection rate (×100,000) of leprosy according to sex, race, age group and illiteracy. Brazil and Regions, 2014‒2019.

Variable/Region	Brazil	North	Northeast	Southeast	South	Midwest
**Sex**						
Male	20.73	47.73	31.23	6.70	4.74	55.39
Female	14.86	31.79	23.21	4.69	2.88	42.60
**Race**						
White	9.01	25.14	15.72	4.48	3.45	36.97
Black and Brown	22.88	41.60	28.53	6.50	4.98	53.16
Yellow	41.58	337.78	158.72	5.37	54.28	70.94
Indigenous	20.54	25.30	20.79	5.90	3.33	105.83
**Age range**						
0 to 14 years	4.28	11.60	7.88	1.04	0.24	6.17
15‒39 years	13.75	34.06	21.01	4.08	2.41	36.28
40‒59 years	25.22	58.97	40.01	11.62	5.79	74.66
60‒79 years	28.14	70.14	53.64	10.04	8.21	77.47
80 or older	18.15	46.67	35.50	5.76	4.88	45.08
**Illiteracy**						
Unknown/white	3.82	7.11	7.16	1.45	0.75	7.91
No (> 15 years)	20.71	48.92	32.87	6.74	5.15	66.53
Yes (> 15 years)	28.62	56.83	28.06	11.97	10.23	80.67

Source: IBGE and DATASUS, adapted by the authors, 2023.

Regarding the clinical characteristics and progression of the disease (Table 3), it is observed that the borderline form (48.48%) is the most common, followed by the lepromatous form (17.59%), with the remaining cases being divided between the indeterminate and tuberculoid forms. The multibacillary operational classification is the most prevalent (75.75%) and incident - 79.55 per 100,000 inhabitants. Regarding bacilloscopy, it was not performed or ignored in 42.80% of cases, 31.48% were negative and 25.72% were positive. As for the dermatological lesions, 36.63% of the patients had more than five skin lesions affected by mycobacteria, with a rate of 38.47 per 100,000 inhabitants; 29.56% had between two and five lesions and 21.41% had only one lesion, corresponding to rates of 31.05 and 22.48, respectively. The absence of lesions (0 reported) and unreported lesions (99 reported) accounted for 12.40% of cases.

**Table 3:** Characteristics of the clinical presentation and leprosy outcome. Brazil, 2014‒2019.

Variable	Frequency (n = 215155)	%	Rate (×100000)
**Clinical Form**			
Indeterminate	25.909	12.04%	12.65
Tuberculoid	27.633	12.84%	13.49
Borderline	104.300	48.48%	50.91
Lepromatous	37.851	17.59%	18.48
Not classified/unknown	19.462	9.05%	9.5
**Operational Classification**			
Paucibacilar	51.870	24.11%	25.32
Multibacilar	162.971	75.75%	79.55
**Bacilloscopy**			
Positive	55.337	25.72%	27.01
Negative	67.729	31.48%	33.06
Not performed/unknown	92.089	42.80%	44.95
**Skin Lesions**			
Informed 0 or 99	26.675	12.40%	13.02
Single lesion	46.056	21.41%	22.48
2 to 5 lesions	63.606	29.56%	31.05
> 5 lesions	78.818	36.63%	38.47
**Type of outcome**			
Cure	166.396	77.34%	81.22
Transferred	18.417	8.56%	8.99
Death	3.552	1.65%	1.73
Abandonment	14.407	6.70%	7.03
Diagnostic error	3.582	1.66%	1.75
Not filled out	8.801	4.09%	4.3
**Grade of disability**			
Grade zero	117.917	54.81%	57.56
Grade I	52.779	24.53%	25.76
Grade II	18.067	8.40%	8.82
Not assessed/unknown	26.392	12.27%	12.88

Source: IBGE and DATASUS, adapted by the authors, 2023.

The majority of the outcomes comprised a cure for the disease (Table 3), with this amount being estimated at 77.34%. Other types of outcomes comprised transfers in 8.56% of the cases and a rate of 8.99; abandonments in 6.70% of the cases and a rate of 7.03; blank in 4.09% of the cases and a rate of 4.3. The occurrences of death and diagnostic error characterized the minority, 1.66% of the cases each, with average rates of 1.74. The grades of physical disability found in 54.81%, 24.53% and 8.40% of the patients at the time of diagnosis were respectively grade 0, 1 and 2, and 12.27% of the cases did not have an assessed grade of disability.

## Discussion

This study aimed to analyze the detection, temporal trend, clinical and epidemiological profile, and performance of social indicators of leprosy in Brazil and its regions. The World Health Organization (WHO) stipulated the elimination of the disease, with a target of less than 1 case per 10,000 inhabitants by 2020.^8^ Despite the slight overall drop in the detection rate in the studied population, only the South and Southeast regions reached the target, even though Brazil proposed the elimination of leprosy as a public health problem three decades ago.^9^

The parameters of the indicators for monitoring progress and elimination of leprosy by the MoH classify the endemicity situation according to the detection rate of new cases as: hyperendemic ≥ 40/100,000 inhab.; very high 20‒39.99/100,000 inhab.; high 10‒19.99/100,000 inhab.; medium 2‒9.99/100,000 inhab.; and low < 2/100,000 inhab.^10^ Thus, one can list the regions from highest to lowest endemicity, starting with the Midwest, classified as hyperendemic with an average detection rate of 48.41/100,000 inhab., followed by the North and Northeast, classified as very high, with 39.62 and 26.95/100,000 inhab., respectively, the Southeast with 5.79/100,000 inhab. and the South with 3.97/100,000 inhab., both regions with average parameters.

The high rates found in the Midwest, North and Northeast regions are due to the difficulty in controlling the disease and late detection of cases,^4^ proven by the main indicator of late diagnosis, physical disability grade II at the time of diagnosis. Of the 18,067 cases diagnosed with this grade, 37.5% occurred in the Northeast region. These results were also found in previous studies.^11^, ^12^ In addition, these regions have the lowest Human Development Indexes (HDI) and the worst variables related to sanitation (proportion of residents in households with rudimentary septic tanks and proportion of residents in households supplied with well water), metrics that show worse living conditions, which are associated with higher disease rates.^13^ Furthermore, the states that comprise the so-called Legal Amazon, an area at risk for leprosy due to the type of land occupation and indexes of living conditions, are all from these regions.^11^

The highest average annual detection rates of new cases of leprosy per 100,000 inhabitants were 48.41 in the Midwest and 39.62 in the North regions, representing figures that are, respectively, 2.77 and 2.26 times higher than the national average for the period. These regions have been considered risk areas for decades^11^ and have been targets of internal migration since 1970, encouraged by the Brazilian government with agricultural modernization and highway construction.^14^ Combined with the overload and lack of infrastructure, leprosy began to gain strength in these regions. The lowest average annual detection rates were found in the South (3.97) and Southeast (5.79) regions, well below the national average of 17.49 cases per 100,000 inhabitants. This finding shows continuity with older studies, whose results also pointed to these peculiarities.^11^, ^12^ The relationship between the disease and socioeconomic conditions is unquestionable. Brazilian studies have already demonstrated a significant relationship between low HDI and the disease in different regions of the country.^13^, ^15^ Thus, as expected, the two most developed Brazilian regions had the lowest rates. However, it is necessary to add indicators such as higher temperature and humidity, greater presence of bacilliferous patients in endemic regions, and genetic susceptibility to factors associated with high disease rates.^16^, ^17^ The regions with the highest rates are the least developed ones, with more fragile urban spaces, population agglomeration, and greater economic vulnerability. Thus, the national situation provides exposure, contamination and persistence of the condition, and also conceives autochthonous and imported cases.^4^

There was a predominance of males, presumably justified by the fact that men are more exposed to risky environments, have greater social contact, and take less care of their health than women, a phenomenon that was observed in other studies on leprosy in Brazil.^18^, ^19^, ^20^ Genetic susceptibility and hormonal interference in the immune response to the bacillus are also factors that are investigated.^21^

There was a predominance of cases in individuals with black skin color, including blacks and browns, in all age ranges analyzed, in agreement with other studies.^21^, ^22^ It is believed that these groups are more affected due to the historical process of social inequality.^22^ The highest detection rate occurred in the Asian ethnic group, 41.58/100,000 inhabitants, showing a higher risk than other ethnic groups. There is no data in the international literature to support this prevalence; therefore, in the present study, the low self-declaration of yellow skin color in the Census is suggested as the probable cause. The Indigenous population has very high rates, which can be linked to the environment, genetics and lifestyle of this population.^23^ It is worth noting the small number of studies focused on this group, is a detrimental scenario for this population, given the significant rates and the fact that the lack of robust analysis delays possible interventions.

Regarding the age range defined by the study, the 40-59 age range had the highest number of cases in total; however, the highest detection rate occurred between the 60-79 years range throughout the country. These data infer that individuals diagnosed with leprosy are part of the economically active population and, as this is a limiting and disabling disease, it can cause harm to the economy. It is also worth noting that the population at greatest risk is the elderly, as shown by the detection rate, which is linked to the long incubation period and the natural decline of the immune system.^21^, ^24^ It is also important to mention that cases in children under 15 years of age are related to at least one confirmed case in the family, with possible pediatric contamination occurring within the family itself due to failure to combat the chain of transmission. It is noteworthy that there is greater difficulty in diagnosis in childhood, culminating in possible disease complications, decreased school performance, and development of the individual due to physical and social impairment.^4^ The detection of leprosy in children under 15 years of age is an indicator of continued transmission of the bacillus,^25^ whose tendency has been decreasing in the regions, being a national trend and therefore indicating a tendency for a decrease in the disease incidence throughout Brazil.

Most affected individuals in raw numbers were literate and the highest detection rate was among illiterate people, a reality in most regions. This discrepancy was even greater in the more developed South and Southeast regions, where the illiteracy rate was approximately twice that of the literate population. Considering that, according to IBGE, only 6.6% of the Brazilian population is illiterate,^26^ the number of illiterate people affected is alarming. This makes them a population at risk, with low levels of schooling and illiteracy being unfavorable social determinants regarding the transmission of Hansen's bacillus and the progression to disabling forms of the disease, as they make it difficult to recognize the clinical manifestations and understand treatment guidelines.^7^, ^19^, ^20^, ^24^^,^^27^

During the analyzed period, the borderline form predominated, accounting for almost half of all cases, followed by the lepromatous form, accounting for 17.59% of the total. These forms were also shown to be dominant in some studies carried out in the North and Midwest regions.^24^, ^28^ Other references indicate the tuberculoid form as the most common, especially in regions where the disease is endemic.^29^ Thus, in Brazil, it can be inferred that the predominant clinical form varies according to the location and its characteristics of endemicity and transmission.

The multibacillary operational classification represented the vast majority of cases (75.75%), in agreement with several studies.^24^, ^28^, ^30^ This finding, combined with the progressive increase in the percentage of multibacillary cases in Brazil since 2010, is especially relevant since individuals classified as multibacillary are important sources of contagion for the community and maintenance of the epidemiological chain.^2^

Of the total number of cases reported throughout Brazil during the period, only 57.2% underwent bacilloscopy, with 45% of the tests being positive, indicating a higher risk of disease transmission.

Regarding skin lesions, 36.63% of the reported cases consisted of patients with more than five lesions; 29.56% had two to five lesions, and 21.41% had a single lesion. This pattern was also found in other studies.^19^, ^29^ The absence of skin lesions and unreported lesions are combined as a single piece of information in the database, equivalent to 12.40%. This association compromises the accuracy of pure neural leprosy frequency, given the impossibility of measuring the absence of skin involvement alone.

The vast majority evolved to cure, which means that these patients correctly completed the polychemotherapy treatment recommended by the Ministry of Health,^2^ with a percentage of 77.34%. Other studies have also shown a high proportion of cured patients in Santa Catarina and Bahia, to the detriment of low death rates.^19^, ^31^ A significant percentage of abandonment (6.7%) stands out, which may be related to the long treatment duration, lack of knowledge about the disease and its progression, skepticism regarding the cure, lack of encouragement, social stigma, leprosy reactions, and also the distance between the patient's residence and the health service.^32^ These patients must be rescued in such a way as to break the transmission chain, resistance to polychemotherapy, and the emergence of physical disabilities.^2^

Most individuals with leprosy did not have sequelae at diagnosis, classified as having grade zero physical disability. This proportion was also present in studies conducted in hyperendemic regions, with very high and medium endemicity,^19^, ^29^, ^30^, ^31^ demonstrating a common distribution throughout the country, with some exceptions that indicate grade I as the most frequent.^24^, ^33^ Approximately 33% of the evaluated cases had grade I or grade II disability at diagnosis, respectively occurring with decreased sensitivity or muscle strength in the hands, feet and/or eyes for grade I patients and visible physical deformities or blindness for grade II patients.^2^ These grades confirm the duration of disease progression, with grade II being evidence of late diagnosis.

The current study had some limitations inherent to its nature. Firstly, the use of secondary data may characterize inconsistencies due to the fact that the notifications were filled out by health professionals who possibly did not receive training or standardized guidelines for filling out the notification forms. Incomplete and “unknown/white” data, especially regarding the items' race/skin color and level of schooling, hinder the epidemiology study. Another issue was the time period assessed, since the data source platform was changed and only has more complete and reliable data from the initial year chosen for the research. It is worth noting that TABNET is a database with continuous updates and may show differences in relation to epidemiological bulletins that have closed data. Additionally, the study could not last longer due to the social isolation imposed by the COVID-19 pandemic, which generated underdiagnosis in the years following its outbreak.

## Conclusion

It can therefore be inferred that leprosy remains a public health problem in Brazil, as evidenced by the maintenance of disease rates in four of the five major regions, with a decrease being observed only in the South region during the studied period. The high rates in the North, Northeast, and Midwest regions pose a risk to the population due to the perpetuated endemicity. Regarding socioeconomic indicators, it was observed that they follow the same trend as described in the literature, with a higher incidence in places with lower HDI, worse living conditions, and type of land occupation.

The results found in the study indicate the need to expand leprosy control measures and intervention strategies at the national level, focusing on the regions with higher endemicity. The National Strategy for Combating Leprosy should be strengthened; the disease and its complications should be addressed and stigma and discrimination should be fought. Based on the analyzed data, it is suggested that emphasis be placed on actions aimed at socioeconomic groups included in the risk profile and that special attention be paid to early diagnosis through the active search for cases aiming at treatment and reduction of transmissibility.

## Financial support

None declared.

## Authors' contributions

Alícia de Siqueira Sczmanski: Approval of the final version of the manuscript; design and planning of the study; drafting and editing of the manuscript; collection, analysis and interpretation of data; effective participation in research orientation; critical review of the literature; critical review of the manuscript.

Lucas Pazin: Approval of the final version of the manuscript; design and planning of the study; drafting and editing of the manuscript; collection, analysis and interpretation of data; effective participation in research orientation; critical review of the literature; critical review of the manuscript.

Thiago Mamoru Sakae: Statistical analysis; approval of the final version of the manuscript; collection, analysis and interpretation of data; effective participation in research orientation; critical review of the manuscript.

Josete Mazon: Approval of the final version of the manuscript; design and planning of the study; effective participation in research orientation; critical review of the manuscript.

## Conflicts of interest

None declared.
